# Test-retest reliability analysis of resting-state EEG measures and their association with long-term memory in children and adults

**DOI:** 10.1162/IMAG.a.1224

**Published:** 2026-04-29

**Authors:** Anastasios Ziogas, Simon Ruch, Nicole H. Skieresz, Sandy C. Marca, Nicolas Rothen, Thomas P. Reber

**Affiliations:** Faculty of Psychology, UniDistance Suisse, Brig, Switzerland; The LINE (Laboratory for Investigative Neurophysiology), Department of Diagnostic and Interventional Radiology, Lausanne University Hospital and University of Lausanne, Lausanne, Switzerland; The Sense Innovation and Research Center, Lausanne and Sion, Switzerland; Faculty of Psychology, University of Geneva, Geneva, Switzerland; Department of Epileptology, University of Bonn Medical Center, Bonn, Germany

**Keywords:** resting-state EEG, long-term memory, alpha power, second language learning

## Abstract

EEG resting-state measures, such as spectral power and microstates, have been associated with human long-term memory (LTM) performance. However, findings across studies are inconsistent and sometimes contradictory, likely due to a low reliability of the measures employed. These inconsistencies limit the interpretability and generalizability of results, emphasizing the need for a systematic evaluation of measure reliability. In this study, we addressed this gap by identifying the most reliable EEG resting-state measures and evaluating their predictive value for LTM performance in a second-language (L2) vocabulary learning paradigm. A group of children (N = 36) and adults (N = 90) participated in two studies on second-language vocabulary learning. Participants completed a test on L2 vocabulary and a resting-state EEG recording (180 seconds eyes open) before and after learning a second language. We used Intraclass Correlation Coefficients (ICC) to identify resting-state EEG measures with satisfying test-retest reliability (ICC > = 0.75) and then assessed how these reliable measures are associated with L2 vocabulary learning representing LTM performance. Highest ICC values were found for oscillatory power in the alpha range and in the frequency of occurrences, duration, and coverages of microstates. Calculations yielded ICC values of 0.84/0.86 (children/adults) for alpha power and 0.88/0.80 for microstate measures. Of these measures, only alpha power showed a positive correlation with LTM performance, but only in the adult population (*r* = 0.38, *p* < .01). No other measures were associated with LTM (all *p* > .05). Alpha power could thus serve as a stable and reliable marker of the neural mechanisms accounting for high LTM performance in the fully developed adult brain.

## 1 Introduction

Our understanding of the neurobiology of human long-term-memory (LTM) is consequential for our conceptualization of the uniqueness of human cognition. Prominent classical cognitive models have often implemented some form of language memory in their framework (Baddeley, 2003) as memory is essential for the development of language (Corballis, 2019). This even led to assumptions on an independently evolved link between verbal working memory and verbal LTM (Hartshorne & Makovski, 2019), further promoted by evidence that LTM is supported by the same neural systems involved in language comprehension and production (Schwering & MacDonald, 2020). Language stimuli or language skills over time represent a useful tool to study LTM with its neurobiological components and EEG is a useful method to capture the temporal dynamics of language processes and their association to LTM.

Task-based EEG measures can be influenced by task demands and performance variability, which reflects their sensitivity to context-dependent cognitive processes. Using such task-based methodology, a link between oscillatory EEG power and LTM has been established (Murphy et al., 2018) even when signals were recorded intracranially (Axmacher et al., 2008). This was also shown when different stimulus modalities were used (e.g., auditory LTM, Lenz et al., 2007). Furthermore, task-based EEG power measures have been shown to be sensitive to both LTM encoding and retrieval (Babiloni et al., 2004). Especially alpha power has been found to correlate with LTM performance (Khader & Rösler, 2011) and activation of semantic concepts (Klimesch, Doppelmayr, Pachinger, & Ripper, 1997; Klimesch, Doppelmayr, Pachinger, & Russegger, 1997). It is assumed that brain activity in the alpha band represents the inhibition of irrelevant mental processes, facilitating memory formation and regulating the flow of information through the brain (Jensen & Mazaheri, 2010). Evidence linking alpha oscillations to long-term memory abilities primarily derives from task-based paradigms, which have provided critical insights into the functional role of alpha dynamics but do not necessarily generalize to resting-state measures.

In resting-state EEG recordings, the participants are not required to perform a task or perceive any stimulation. The minimal demands placed on participants make this approach well suited for studying populations such as children. This line of research has provided heterogeneous findings on LTM or language within different developmental stages (e.g., Lui et al., 2021). This could be partly due to the multitude of methodological approaches available to handle resting-state EEG data, for example, calculations of EEG power, microstates. The most frequently used measures and parameters in the field of resting-state EEG are the absolute or relative oscillatory power for the prominent frequency ranges (e.g., delta: 0.5–4 Hz). Resting-state oscillatory power has been linked directly to LTM (Brokaw et al., 2016), long-term potentiation (Savarimuthu & Ponniah, 2024), and memory consolidation during sleep, also when recorded intracranially (Marshall et al., 2006). A decrease in alpha power during rest was also demonstrated through sleep deprivation (24 and 36 hours) (Lian et al., 2023), and sleep has been suggested as an essential process for LTM formation (Klinzing et al., 2019). In a recent study, resting-state EEG power was used to successfully predict semantic LTM formation after 2, 4, and 6 months, outperforming other known behavioral predictors such as Raven’s Advanced Progressive Matrices (Amin et al., 2025), which were interpreted by the authors as indirect indicators of LTM. Overall, resting-state EEG power has been associated with long-term memory processes and has been interpreted as reflecting trait-like neural characteristics. At the same time, the role of specific frequency bands, and alpha power in particular, remains unresolved. This uncertainty is also evident in resting-state studies focusing on language-related abilities.

Early works on resting-state EEG power and language have shown lower theta (Colon et al., 1979; Sklar et al., 1972) and higher alpha power (Duffy et al., 1980) in small samples of healthy children compared to dyslexic children. More significant changes in these frequency bands were observed over a period of 2.5–3 years in poor readers compared to typical readers (Harmony et al., 1995). This was interpreted as evidence of maturational delay in readers with impairments. The findings in the theta range were replicated in later studies on children (Arns et al., 2007) and also in adult dyslexics (Rumsey et al., 1989). Low theta power localized in the left hemisphere was also related to higher sentence comprehension (Beese et al., 2017). The pattern of diminished low frequency (delta, theta) power and pronounced higher frequency (alpha, beta, gamma) power was often related to better language function throughout the literature (Barry et al., 2009; Benasich et al., 2008; Brito et al., 2016; Clarke et al., 2002; Gou et al., 2011; Lum et al., 2022; Papagiannopoulou & Lagopoulos, 2016; Tierney et al., 2014) while some studies did not replicate this pattern (Babiloni et al., 2012; Lui et al., 2021; Nazari et al., 2012; Pinkerton et al., 1989; Schiavone et al., 2014; Shiota et al., 2000). When EEG-neurofeedback was used to train a group of children with reading disabilities, improvement in reading was not related to any changes in resting-state-EEG power (Nazari et al., 2012).

The diversity of findings may be partly explained by methodological differences or differences in stability of specific power measures. Resting-state EEG activity is commonly interpreted as reflecting intrinsic properties of large-scale brain organization and has, therefore, been proposed to capture temporally stable, trait-like neural characteristics (Sadaghiani & Kleinschmidt, 2013). Accordingly, simple power-based analyses of scalp EEG—particularly during resting states—have been widely applied to examine individual differences in long-term memory and language-related abilities across time. Establishing the stability and reliability of these measures is a critical prerequisite for evaluating their suitability as neural markers of individual differences and for interpreting associations with longitudinal changes in LTM performance.

Less common is the calculation of so-called microstates or estimations about the cortical sources of EEG signals generated on the scalp. Microstate analyses have the advantage that they rely on multivariate data-driven methods (Michel & Koenig, 2018) that do not require predefining specific frequencies, time windows, or scalp regions of interest. Microstates are unique electric fields or voltage topographies in the scalp EEG that repeatedly occur for brief, quasi-stable periods (Lehmann et al., 1987; Strik & Lehmann, 1993). Different states are thought to represent distinct neuronal generators and mechanisms in the brain (Lehmann et al., 1987; Strik & Lehmann, 1993). Microstates also rely on relatively simple computation methods (Khanna et al., 2015) producing reliable results (Kleinert et al., 2023) and resting-state microstates have been linked directly to memory consolidation of word-pairs (Poskanzer et al., 2021). Usually, a set of prototypical (e.g., four) microstates are identified in the recorded segment and used to label the entire resting-state recording into sequences of the prototypical microstates. Several statistics (e.g., Global Field Power, frequency of occurrence) can then be estimated for every microstate and used for further analysis. When looking at microstates at the scalp, one study reported a negative correlation between the resting-state microstate class E and a language test (Mini Mental State Examination subtest) (Grieder et al., 2016). Right-lateralized sources of resting-state microstate class B were related to verbal processing in another study (Milz et al., 2016). Resting-state EEG microstates have been linked to large-scale brain networks and trait-like cognitive functions. Jann et al. (2010) showed that canonical microstates correspond to resting-state networks observed in fMRI, such as the default mode and frontoparietal control networks. Other studies reported associations between resting-state microstate dynamics and sustained attention or working memory (Jabès et al., 2021; Seitzman et al., 2017), while atypical resting-state microstate patterns have been found in individuals with developmental disorders (Tomescu et al., 2014). These findings support the use of resting-state microstates as stable neural markers suitable for investigating individual differences in language learning and LTM. By exploring the reliability of these measures within a longitudinal design, their potential as cognitive biomarkers can be evaluated in a real-world learning context.

Some studies have analyzed the reliability of resting-state EEG power and microstates (e.g., Lopez et al., 2023; Popov et al., 2023); however, these measures have not yet been directly linked to LTM performance. The aim of this study is to assess the reliability of resting-state EEG power and microstate measures at the scalp level and, based on these results, explore whether the most reliable measures are associated with LTM performance, while accounting for developmental effects. High split-half and test-retest reliability are essential prerequisites for using a measure as a potential marker of the neural architecture and processes underlying LTM performance, as they ensure the consistency and stability necessary for meaningful interpretation. Within a longitudinal study on second language acquisition using an educational app for Swiss schoolchildren and adults, the experimental design already provides an optimal opportunity to quantify split-half and test-retest reliability for the candidate resting-state measures and directly relate them to language improvement. This study involves participants from two age groups completing a resting-state EEG session and a language test at two timepoints, with second-language vocabulary learning occurring between sessions through a combination of instructional methods (e.g., classroom instruction, traditional study materials, and, where applicable, a language-learning app). Using this setting, the collected data at both timepoints can be explored in a strictly data-driven and hypothesis-free manner to address the question posed above. Results gained from this research could be of particular interest for future studies in this field and also help to navigate through already existing inconsistencies in the literature.

## 2 Methods

### 2.1 Study overview and procedure

As part of a larger longitudinal study on language learning, two age groups were recruited to participate in consecutive experimental sessions involving multiple data collection timepoints. These two studies included a group of children in one study and a group of adults in the other. Both groups underwent various cognitive and neurophysiological tests over time and engaged in second-language (L2) vocabulary learning between testing sessions through different instructional means (e.g., classroom instruction, traditional study materials, and, where applicable, a language-learning app). Participants who fully completed two vocabulary tests and two resting-state EEG recordings were included in the test–retest reliability analyses for the present two studies. Between the two timepoints (T1 and T2) for vocabulary tests and resting-state EEG sessions, participants were encouraged to use the provided language-learning app or other available means (e.g., classroom instruction or traditional study materials) to study a second language. Consequently, both vocabulary tests and resting-state EEG sessions were scheduled to assess behavioral performance and neurophysiological changes before (T1) and after (T2) the language learning period. Progress in language learning and corresponding changes in LTM performance were anticipated during this interval. The children group and the adult group were treated as separate studies. EEG power values and microstate measures served as the dependent variables.

### 2.2 Study 1

#### 2.2.1 Participants

All child participants were recruited in the German and French-speaking regions of Switzerland, with native languages being either French or German. To ensure the validity of the results, only healthy participants, free from any chronic medical conditions, psychological disorders, language or memory deficits, were included in the study. A total of 44 children took part in the study. Of these, 36 participants (18 females, 23 righthanded, age M = 11.22 years, SD = 1.16, range = 9–15) fully completed the two testing sessions without technical problems and with the adequate data quality required for the present reliability analysis. All participants’ parents signed informed consent (study approved by UniDistance Suisse ethics committee; approval nr.: 2019-12-00002).

#### 2.2.2 Materials & procedure

We employed a one-factorial within-subjects design with time (pre [T1]/post [T2] intervention) as the single factor. The dependent variables included performance from the vocabulary test, EEG power within the standard frequency bands, as well as variables derived from microstate analysis. This design allowed us to examine changes in both behavior and neurophysiological activity across the two time points, providing insights into the effects of the intervention. Each child initially completed a vocabulary test (T1), typically administered in a paper-and-pencil format within a classroom setting. This was followed by an EEG recording session (T1), conducted either on the same day or the following day. After completing T1, children were encouraged to use the provided language-learning app or other available means—such as regular classroom instruction, textbooks, and traditional study materials—to study their second language. Participants were either native German (25) or French (11) speakers (L1) and learned vocabulary in their respective second language (L2), either French or German. Given that L2 is an official language and part of the standard school curriculum, children were naturally exposed to it through multiple learning channels during the study period. The vocabulary tests were tailored accordingly, assessing French or German vocabulary depending on each child’s L1. The frequency and intensity of app usage were self-directed and varied across participants. Following the learning period, the children completed a second vocabulary test and EEG session (T2).

The vocabulary test was a cued-recall translation task comprising 20 items. Half of the items required translation from L1 to L2, while the other half required translation from L2 to L1. Participants provided their responses in written form. Responses on the translation items within a vocabulary test were marked as either correct or incorrect, and the result was transformed into a percentage score representing behavioral performance. This design allows for the calculation of difference scores (T2 minus T1), representing an approximation to changes in LTM performance. Regardless of the individual app usage and language aptitude, each participant’s vocabulary test difference score (T2 minus T1) can be related to neurophysiological parameters, that show high reliability in our analysis. The percentage scores representing improvement in L2 were used as a proxy for LTM performance. These scores were correlated with EEG resting-state measures at T1. This analysis aimed to estimate the predictive value of the stable neurophysiological markers at T1 for changes in LTM performance over time, as evidenced by the improvement in behavioral performance in L2 within the vocabulary test.

Participants sat in a chair with their eyes open and were told to fixate on a cross on a screen while the resting-state EEG was recorded for 180 seconds. EEG was recorded using a 64-channel sponge-based RNet system (Brain Products GmbH), arranged according to the extended international 10–20 system (ground: FPz, reference: Cz), with electrode impedances kept below 30 kΩ. Signals were digitized at a sampling rate of 1,000 Hz via a BrainAmp amplifier and recorded on a Lenovo laptop (Windows 10 Home, 8 GB RAM). The 180-second-long eyes-open segments were pre-processed with the EEGLAB toolbox (v2023.0) for MATLAB (R2020b). The data were re-referenced to the common average and was filtered with a high-pass (0.5 Hz) and a low-pass (80 Hz) filter. Line-noise was removed at 50 Hz and its harmonics up to 150 Hz using the function ‘pop_cleanline()’ with default settings. Artifact rejection and removal of bad channels was performed using the function ‘pop_clean_rawdata()’ with standard parameters. Eye-movement artifacts and muscle artifacts were removed using Independent Component Analysis (ICA). ICA was performed on a copy of the data that was bandpass-filtered between 1–30 Hz and was then down-sampled to 100 Hz. Artifact-laden components were identified using ‘pop_iclabel()’ with default threshold settings (Pion-Tonachini et al., 2019). All components related to eye-movements and muscle artifacts were removed from the original data. Finally, previously rejected channels were recovered using spherical interpolation, and all channels were again re-referenced to the common average.

#### 2.2.3 Data analysis

The data were analyzed in a data-driven, hypothesis-free manner. This approach allows for an unbiased examination of the stability of the different measures. In general, data points outside 2 SD of the mean were identified as outliers, and the corresponding participants were excluded from the respective statistical analyses, resulting in minimally varying sample sizes (and df) across tests. In all correlational analyses throughout the present studies, Bonferroni correction was applied to the p-values for all the correlations conducted to account for multiple (21) comparisons. All statistical analyses were conducted using R version 4.3.1 (R Core Team, 2023).

##### 2.2.3.1 EEG power

The EEGLAB function ‘spectopo()’ was used to obtain the average power spectral density (PSD) for 2-second epochs with an 80% overlap over the entire length of resting-state recording (180 seconds), ensuring a detailed frequency resolution. This analysis provided a frequency resolution of 0.5 Hz, covering a frequency range of 0.5 Hz to 80 Hz. The output from ‘spectopo()’ was converted from decibels (dB) to a linear scale (µV²/Hz) to facilitate further analysis. For each electrode, the absolute power within five standard frequency bands (delta: 1–4 Hz, theta: 4–8 Hz, alpha: 8–14 Hz, beta: 14–30 Hz, gamma: 30–75 Hz) was calculated over the specified frequency range. For each frequency band, the corresponding indices in the frequency vector were identified. The power within the band was then calculated by taking the integral of the PSD over the specified frequency range using the trapezoidal rule, which provides an estimate of the area under the curve of the power spectrum within that band. This process effectively quantifies the total power contained in each frequency band of the EEG signal.

Additionally, relative power for each frequency band was determined by normalizing the absolute power in each band to the total power across all frequencies resulting in a percentage value. Finally, to obtain a comprehensive measure of brain activity, the absolute and relative powers were averaged across all electrodes for each frequency band. This approach provided an overall representation of the brain’s spectral power distribution.

For subsequent statistical analysis, the absolute power values for each frequency band were log-transformed using the base-10 logarithm. This transformation was performed to normalize the data distribution and stabilize variance, which are prerequisites for parametric statistical tests.

##### 2.2.3.2 Microstates

To calculate microstates, time points of interest for the analysis are typically identified using peaks in global field power (GFP) which represent pronounced EEG scalp topography. Such quasi-stable states of the electric field are assumed to last for short moments (60–120 ms) and presumably relate to differentiable functional states of the brain (Wackermann et al., 1993). Microstate analyses were done using the EEGLAB plugin MICROSTATELAB by Koenig (Nagabhushan Kalburgi et al., 2023). The ‘pop_FindMSMaps()’ function was used on the pre-processed EEG files to identify individual template maps (4–7 class number solutions in the k-means algorithm). Polarity was ignored, 20 restarts were used per dataset, and the clustering was completed on all GFP peaks. Four mean microstate maps across a single group within a time point (e.g. children T1) were identified using the ‘pop_CombMSMaps()’ function and four grand mean maps were identified across all means while ignoring polarity (Koenig et al., 2002). The solution of four classes of grand mean microstate maps was used to backfit to the individual EEG data sets with the function ‘pop_FitMSMaps()’ as four maps are considered an optimal number for such analyses (Michel & Koenig, 2018). The mean duration, occurrence, coverage (fraction of total recording time that the microstate is dominant), and mean GFP of the four classes were calculated at the subject level and used for further statistical analysis.

##### 2.2.3.3 Reliability analysis

To measure the reliability of the EEG resting-state measures between sessions, test-retest reliability was estimated using T1 and T2 measures. Interclass Correlation Coefficients (ICC) (Fisher, 1970) were calculated to quantify reliability, utilizing the R-package ‘psych’ (Revelle, 2023). ICCs were computed using the ICC(2,1) a two-way random-effects model for absolute agreement between measurements. This approach is appropriate when both subjects and measurement occasions are considered random samples and when absolute agreement is of interest (Shrout & Fleiss, 1979). The formula for ICC(2,1) is:

$$\text{ICC(2,1)}=\frac{MS_{B}\text{​}-MS_{E}}{MS_{B}\text{​}+\left(k-1\right)MS_{E}\text{​}+\frac{k\left(MS_{R}\text{​}-MS_{E}\right)}{n}}$$

*MS_B_* = Mean Square Between subjects

*MS_R_* = Mean Square Between raters (e.g., sessions)

*MS_E_* = Residual/Error Mean Square

*k* = Number of raters (here, sessions)

*n* = Number of subjects

ICC values range from 0 to 1, with higher values indicating greater reliability. Specifically, values from 0.5–0.75 indicate moderate reliability, values from 0.76–0.9 indicate good reliability, and values above 0.9 represent excellent reliability (Koo & Li, 2016). Significance tests further determine if the ICC is significantly different from 0 (alternative hypothesis).

To estimate the within-session stability of the relevant EEG resting-state measures, split-half reliability measures were calculated. The 180-second-long raw segments from both T1 and T2 were divided into two 90 second segments each. Test-retest reliability was assessed using ICC values derived from two 180-second segments, while split-half reliability was calculated using the 90-second segments. The measures from the two 90-second segments within each timepoint (T1 and T2) were correlated using Spearman-Brown-Correction (Lopez et al., 2023). This method provides an additional estimate of split-half reliability for both T1 and T2, complementing the test-retest reliability of all relevant resting-state measures. A measure was considered sufficiently reliable if both the split-half and test-retest reliability were equal to or exceeded 0.75. According to Koo and Li (2016), values between 0.75 and 0.90 indicate good reliability. This threshold was applied consistently to both test–retest ICCs and Spearman–Brown–corrected split-half correlations, as both reflect estimates of measurement stability across time or data segments. Reliability metrics (ICC(2,1) and Spearman–Brown–corrected split‑half) were computed without covariates.

### 2.3 Study 2

Unless otherwise specified, study 2 was conducted in the same manner as study 1.

#### 2.3.1 Participants

Out of 96 recruited participants, a total of 90 adults (58 females, 84 righthanded, age M = 28.96, SD = 6.85, range = 18–40) completed all required testing sessions without issues. All participants gave informed consent (study approved by UniDistance Suisse ethics committee; approval nr.: 2019-12-00002). Participants were either native German (48) or French (42) speakers and used the app to learn basic Finnish vocabulary. Due to organizational and logistic reasons, vocabulary tests and resting-state EEG sessions could not always be scheduled on the same day for the adults. Some participants, therefore, completed T1 vocabulary and EEG recording in more than one session before starting the app usage.

#### 2.3.2 Material & procedure

Adults used the app to practice Finish as a second language. The vocabulary tests were designed accordingly, with adults being tested on Finish material, with more items (48) and with computerized versions of the vocabulary test. Adults used the app following a more structured and consistent schedule, either for 1 hour per day over 2 weeks or approximately 3 hours every third day over 3 weeks. The training protocol consisted of 48 initial encoding trials, 12 daily sessions of 48 trials each, and a final assessment session of 20 trials (total = 644 trials per participant). Compliance was regularly monitored, and participants were reminded when sessions were missed, ensuring comparable training intensity across all individuals.

## 3 Results

### 3.1 Study 1

Children took the vocabulary tests on average 71.33 days (SD = 26.12) apart and completed the EEG resting-state recordings 59.69 days (SD = 22.08) apart.

#### 3.1.1 Behavioral measures

**Fig. 1.**
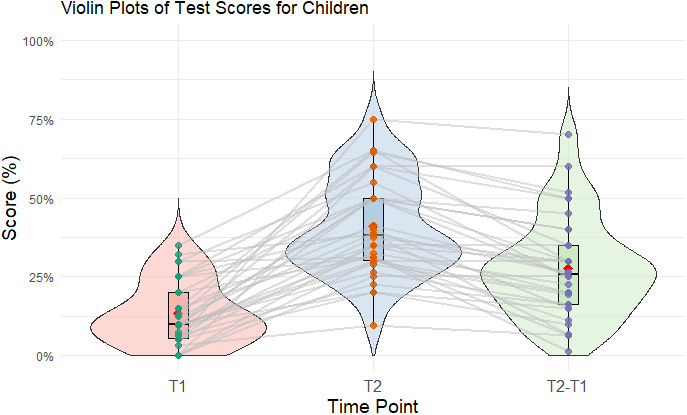
Violin plots for the performance on the vocabulary test in the children sample of study 1. The plots show the mean (red), the median (horizontal line), the boxplot, the interquartile range (vertical line), and the density distribution of the data at each timepoint (T1, T2 and T2 minus T1).

Figure 1 shows the results from the vocabulary test. At T1, children performed with an average score of 14.51% (SD = 11.04%) and improved to 42.16% (SD = 16.90%) at T2 after training. Children’s test scores at T1 were above zero, consistent with their prior familiarity with the language (one-sample t-test: *t*(35) = 7.88, *p* < .001). The difference score (T2 minus T1) of 27.65% (SD = 17.49%) represents the changes in LTM performance based on improvement in L2 within the children sample. A paired-samples t-test showed a significant difference between T1 and T2 *t*(33) = −9.98, *p* < .001, *d* = 2.07.

#### 3.1.2 EEG measures

##### 3.1.2.1 EEG power

Table 1 shows the ICC results for the children sample in our study 1 for both absolute and relative power calculations. While all ICC values in the present study were significantly greater than 0 at the 5% alpha level only the bold numbers represent good reliability according to guidelines (ICC > = 0.75). The overall high split-half reliability calculations are also listed separately for T1 and T2.

**Table 1.** EEG power reliabilities in the children sample (study 1).

	Test-retest reliability		Split-half reliability	
	ICC (95% CI)		T1	T2
Power measure	Children study 1	*p* (ICC)	Children study 1	
Absolute				
Delta	**0.77 [0.58, 0.88]**	<.001	**0.96**	**0.97**
Theta	**0.75 [0.55, 0.87]**	<.001	**0.97**	**0.94**
Alpha	**0.81 [0.65, 0.90]**	<.001	**0.94**	**0.94**
Beta	**0.76 [0.57, 0.87]**	<.001	**0.98**	**0.94**
Gamma	0.54 [0.26, 0.74]	<.001	**0.87**	**0.88**
Relative				
Delta	**0.81 [0.66, 0.90]**	<.001	**0.89**	**0.91**
Theta	0.61 [0.35, 0.79]	<.001	**0.89**	**0.94**
Alpha	**0.84 [0.71, 0.92]**	<.001	**0.92**	**0.96**
Beta	**0.80 [0.64, 0.90]**	<.001	**0.97**	**0.98**
Gamma	0.59 [0.33, 0.77]	<.001	**0.88**	**0.93**

*Note*. Bold ICC values represent good test-retest reliability (> = 0.75) in the present study. Values in brackets represent the 95% confidence intervals for the ICC estimates. Split-half reliability was calculated with the Spearman-Brown-Correction (between the two halves of 90 seconds segments). Bold split-half reliability values represent good split-half reliability (> = 0.75). n. sig. *p* > .05

##### 3.1.2.2 Microstates

**Fig. 2.**
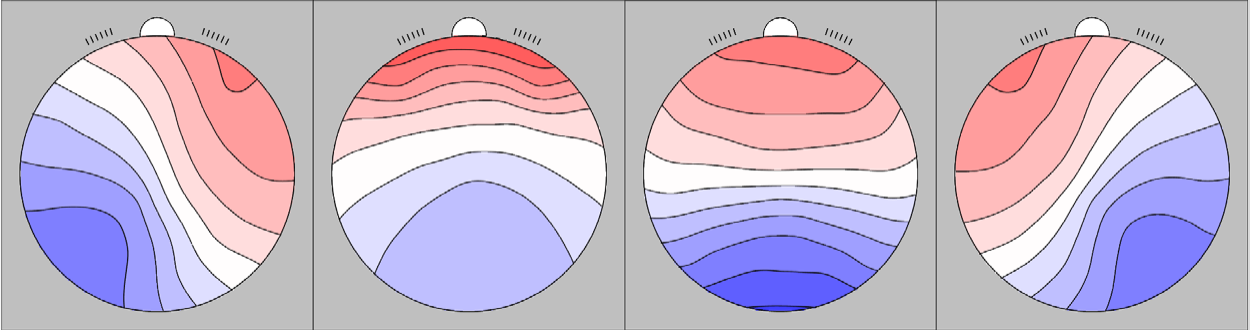
Grand mean microstate maps (A, B, C, D, displayed from left to right) identified in the present study.

Figure 2 shows the four grand mean microstate maps when data from both studies are combined. Table 2 displays the reliability estimates for the microstate measurements (mean GFP, duration, occurrence, duration and coverage of the four classes) in the children sample of study 1. Split-half reliability and ICC values are overall smaller for microstate measures than for EEG power. Except for one ICC value (microstate B coverage: ICC(2,1) = 0.28, 95% CI [–0.06, 0.56], F(32, 32) = 1.78, *p* = .054), all microstate measures in the present study showed an ICC significantly greater than 0 at the 5% alpha level.

**Table 2.** EEG microstates reliabilities in the children sample (study 1).

	Test-retest reliability		Split-half reliability	
	ICC (95% CI)		T1	T2
Microstates measure	Children study 1	*p* (ICC)	Children study 1	
**A**				
GFP	0.72 [0.50, 0.85]	<.001	**0.94**	**0.95**
Occurrence	0.33 [0.00, 0.60]	=.025	**0.87**	**0.72**
Duration (ms)	0.71 [0.50, 0.85]	<.001	**0.86**	**0.88**
Coverage (%)	0.53 [0.23, 0.74]	<.001	**0.81**	**0.73**
**B**				
GFP	0.63 [0.38, 0.80]	<.001	**0.96**	**0.96**
Occurrence	**0.80 [0.63, 0.90]**	<.001	**0.90**	**0.89**
Duration (ms)	0.63 [0.37, 0.80]	<.001	**0.87**	**0.84**
Coverage (%)	0.28^n.sig^ [-0.06, 0.56]	=.054	**0.89**	**0.81**
**C**				
GFP	0.63 [0.38, 0.80]	<.001	**0.97**	**0.97**
Occurrence	**0.79 [0.62, 0.89]**	<.001	**0.92**	**0.85**
Duration (ms)	0.49 [0.19, 0.71]	=.001	**0.85**	**0.86**
Coverage (%)	0.38 [0.06, 0.63]	=.010	**0.97**	**0.91**
**D**				
GFP	0.71 [0.50, 0.85]	<.001	**0.96**	**0.96**
Occurrence	0.72 [0.51, 0.85]	<.001	**0.95**	**0.93**
Duration (ms)	**0.88 [0.78, 0.94]**	<.001	**0.91**	**0.85**
Coverage (%)	**0.83 [0.69, 0.91]**	<.001	**0.97**	**0.92**

*Note*. Bold ICC values represent good test-retest reliability (> = 0.75) in the present study. Values in brackets represent the 95% confidence intervals for the ICC estimates. Split-half reliability was calculated with the Spearman-Brown-Correction (between the two halves of 90 seconds segments). Bold split-half reliability values represent good split-half reliability (> = 0.75). n. sig. *p* > .05

#### 3.1.3 Correlational analyses

Focusing only on sufficiently reliable power measures (both test-retest and split-half reliability > = 0.75), all correlations (Pearson) were calculated between the reliable power measures at T1 (7 measures of EEG power) and the change in LTM performance between T1 and T2 in the children sample of study 1. The results (Bonferroni corrected) are shown in Table 3. There was no significant correlation in this analysis (all *p* > .05).

**Table 3.** Correlations between reliable EEG power measures and LTM in the children sample (study 1).

Absolute	Delta	Theta	Alpha	Beta
LTM	0.19	0.16	-0.15	0.08
Relative	Delta	-	Alpha	Beta
LTM	0.25	-	-0.36	-0.05

*Note*. Correlations of reliable EEG power measures at T1 and LTM measures (vocabulary test performance T2-T1 in %). *p < .05, **p < .01 (Bonferroni correction for multiple comparisons). Correlations are not reported for EEG power measures with insufficient reliability.

Focusing only on sufficiently reliable microstate measures (both test-retest and split-half reliability > = 0.75), all correlations (Pearson) were calculated between the reliable power measures at T1 (4 measures of EEG power) and the change in LTM performance between T1 and T2 in the children sample of study 1. The results (Bonferroni corrected) are shown in Table 4. There was no significant correlation in this analysis (all *p* > .05).

**Table 4.** Correlations between reliable EEG microstates measures and LTM in the children sample (study 1).

Microstate	B occurrence	C occurrence	D duration	D coverage
LTM	-0.09	-0.13	0.00	0.05

*Note*. Correlations of reliable EEG microstate measures at T1 and LTM measures (vocabulary test performance T2-T1 in %). *p < .05, **p < .01 (Bonferroni correction for multiple comparisons). Correlations are not reported for EEG microstate measures with insufficient reliability.

##### 3.1.3.1 Age-adjusted EEG-behavior associations

To assess potential effects of children’s developmental stages on our overall findings, we conducted additional age-adjusted analyses focusing on absolute and relative alpha power, the only EEG measures showing relevant effects in the primary analyses. In the child sample, linear regression models revealed that, after controlling for age, absolute alpha power was not significantly associated with changes in LTM performance, *β* = −0.01, *t*(28) = −1.72, *p* = .096, 95% CI [−0.02, 0.00]. Age showed a significant negative association with LTM change in this model, *β* = −0.06, *t*(28) = −2.28, *p* = .031. Similarly, when controlling for age, relative alpha power was not significantly associated with LTM change, *β* = −0.98, *t*(28) = −1.85, *p* = .075, 95% CI [−2.07, 0.11], and age was not a significant predictor in this model, *β* = −0.05, *t*(28) = −1.70, *p* = .100.

### 3.2 Study 2

In the adult sample, the two sessions with the vocabulary test were on average 29.86 days (SD = 28.09) apart and 14.13 days (SD = 0.77) elapsed between the first and the second EEG resting-state recordings.

#### 3.2.1 Behavioral measures

**Fig. 3.**
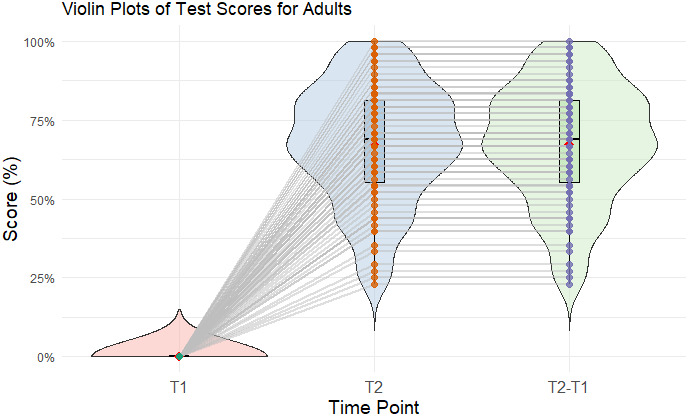
Violin plots for the performance on the vocabulary test in the adult sample of study 2. The plots show the mean (red), the median (horizontal line), the boxplot, the interquartile range (vertical line), and the density distribution of the data at each timepoint (T1, T2, and T2 minus T1).

Adults showed a significant improvement in LTM performance from T1 to T2. This was indicated by a change in vocabulary test performance of 65.13% (SD = 21.75%) with an average of 0.00% (SD = 0.50%) at T1 and an average of 65.13% (SD = 21.76%) at T2. A paired-samples t-test showed a significant difference between T1 and T2 *t*(82) = −32.66, *p* < .001, *d* = 5.06 (Fig. 3).

#### 3.2.2 EEG measures

##### 3.2.2.1 EEG power

Table 5 shows the ICC results for the adult sample in our study 2 both for absolute and relative power calculations. Overall, the split-half reliabilities in this sample were very high and the highest ICC values were observed in the alpha band. Specifically, absolute alpha power showed an ICC(2,1) of 0.88, 95% CI [0.82, 0.92], F(85, 85) = 15.17, *p* < .001, and relative alpha power showed an ICC(2,1) of 0.86, 95% CI [0.79, 0.91], F(84, 84) = 13.76, *p* < .001.

**Table 5.** EEG power reliabilities in the adult sample (study 2).

	Test-retest reliability		Split-half reliability	
	ICC (95% CI)		T1	T2
Power measure	Adults study 2	*p* (ICC)	Adults study 2	
Absolute				
Delta	0.66 [0.51, 0.76]	<.001	**0.96**	**0.93**
Theta	**0.77 [0.66, 0,85]**	<.001	**0.95**	**0.97**
Alpha	**0.88 [0.82, 092]**	<.001	**0.97**	**0.98**
Beta	**0.81 [0.72, 0.87]**	<.001	**0.98**	**0.98**
Gamma	**0.75 [0.64, 0.83]**	<.001	**0.95**	**0.95**
Relative				
Delta	**0.75 [0.64, 0.83]**	<.001	**0.93**	**0.87**
Theta	**0.80 [0.71, 0.87]**	<.001	**0.91**	**0.93**
Alpha	**0.86 [0.79, 0.91]**	<.001	**0.94**	**0.95**
Beta	**0.79 [0.70, 0.86]**	<.001	**0.95**	**0.97**
Gamma	0.64 [0.49, 0.75]	<.001	**0.92**	**0.96**

*Note*. Bold ICC values represent good test-retest reliability (ICC > = 0.75) in the present study. Values in brackets represent the 95% confidence intervals for the ICC estimates. Split-half reliability was calculated with the Spearman-Brown-Correction (between the two halves of 90 seconds segments). Bold split-half reliability values represent good split-half reliability (> = 0.75). n. sig. *p* > .05

##### 3.2.2.2 Microstates

Table 6 displays the reliability estimates for the microstate measurements (mean GFP, duration, occurrence, duration, and coverage of the four classes) in the adult sample of study 2. Split-half reliabilities and ICC values are overall smaller for microstate measures than for EEG power. All microstate measures in the present study showed an ICC significantly greater than 0, the 5% alpha level. However, only two ICC values (bold) represent good reliability according to guidelines. Specifically, microstate D duration showed an ICC(2,1) of 0.80, 95% CI [0.71, 0.87], F(85, 85) = 9.15, *p* < .001, and microstate D coverage showed an ICC(2,1) of 0.79, 95% CI [0.70, 0.86], F(84, 84) = 8.70, *p* < .001.

**Table 6.** EEG microstates reliabilities in the adult sample (study 2).

	Test-retest reliability		Split-half reliability	
	ICC (95% CI)		T1	T2
Microstates measure	Adults study 2	*p* (ICC)	Adults study 2	
A				
GFP	0.71 [0.59, 0.80]	<.001	**0.95**	**0.96**
Occurrence	0.66 [0.52, 0.77]	<.001	**0.85**	**0.89**
Duration (ms)	0.68 [0.54, 0.78]	<.001	**0.86**	**0.92**
Coverage (%)	0.55 [0.38, 0.68]	<.001	**0.81**	**0.90**
B				
GFP	0.71 [0.58, 0.80]	<.001	**0.97**	**0.97**
Occurrence	0.57 [0.41, 0.70]	<.001	**0.87**	**0.95**
Duration (ms)	0.63 [0.48, 0.74]	<.001	**0.86**	**0.93**
Coverage (%)	0.38 [0.18, 0.55]	<.001	**0.85**	**0.92**
C				
GFP	0.66 [0.51, 0.76]	<.001	**0.94**	**0.96**
Occurrence	0.68 [0.54, 0.78]	<.001	**0.92**	**0.95**
Duration (ms)	0.59 [0.43, 0.72]	<.001	**0.79**	**0.93**
Coverage (%)	0.59 [0.43, 0.71]	<.001	**0.84**	**0.91**
D				
GFP	0.71 [0.58, 0.80]	<.001	**0.97**	**0.97**
Occurrence	0.74 [0.63, 0.82]	<.001	**0.92**	**0.95**
Duration (ms)	**0.80 [0.71, 0.87]**	<.001	**0.93**	**0.94**
Coverage (%)	**0.79 [0.70, 0.86]**	<.001	**0.96**	**0.95**

*Note*. Bold ICC values represent good test-retest reliability (ICC > = 0.75) in the present study. Values in brackets represent the 95% confidence intervals for the ICC estimates. Split-half reliability was calculated with the Spearman-Brown-Correction (between the two halves of 90 seconds segments). Bold split-half reliability values represent good split-half reliability (> = 0.75). n. sig. *p* > .05

#### 3.2.3 Correlational analyses

**Fig. 4.**
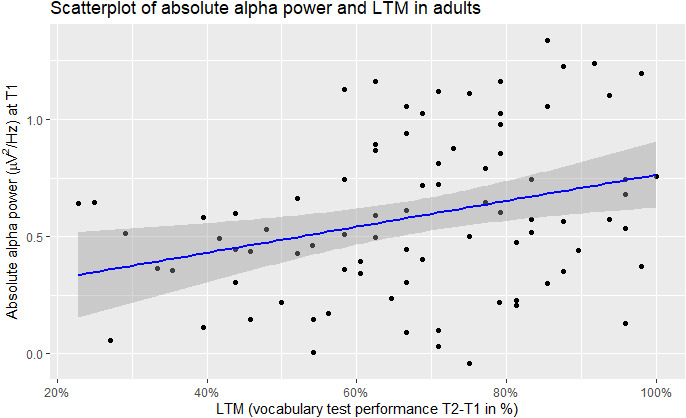
Scatterplot of absolute alpha power (%) and LTM (vocabulary test performance T2-T1 in %) for the adult sample of study 2.

**Fig. 5.**
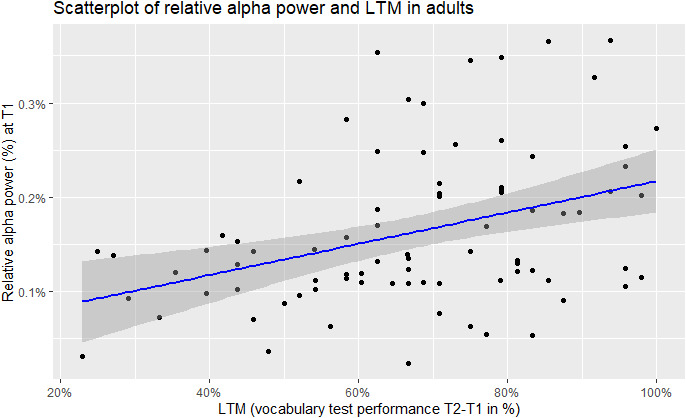
Scatterplot of relative alpha power (%) and LTM (vocabulary test performance T2-T1 in %) for the adult sample of study 2.

Focusing only on sufficiently reliable power measures (> = 0.75), all correlations (Pearson) were calculated between the reliable power measures at T1 (8 measures of EEG power) and the LTM in the adult sample of study 2. The results are shown in Table 7. Alpha power values were significantly positively correlated with LTM performance in the adult sample. Absolute alpha power showed a moderate positive correlation with LTM improvement, r(81) = 0.31, 95% CI [.10, .49], *p* = .004, Bonferroni-adjusted *p* = .044. Relative alpha power showed a similar association, r(80) = 0.38, 95% CI [.18, .55], *p* < .001, Bonferroni-adjusted *p* = .004. A scatterplot for the significant correlations is depicted in Figures 4 and 5.

**Table 7.** Correlations between reliable EEG power measures and LTM in the adult sample (study 2).

Absolute	-	Theta	Alpha	Beta	Gamma
LTM	-	0.07	0.31*	-0.09	-0.24
Relative	Delta	Theta	Alpha	Beta	-
LTM	0.02	0.12	0.38**	0.05	-

*Note*. Correlations of reliable EEG power measures at T1 and LTM measures (vocabulary test performance T2-T1 in %). *p < .05, **p < .01 (Bonferroni correction for multiple comparisons). Correlations are not reported for EEG power measures with insufficient reliability.

Again, focusing only on sufficiently reliable microstate measures (> = 0.75) all correlations (Pearson) were calculated between the reliable microstate measures at T1 (2 microstate measures) and the LTM performance in the adult sample of study 2. The results are shown in Table 8. No correlations were significant (all *p* > .05).

**Table 8.** Correlations between reliable EEG microstates measures and LTM in the adult sample (study 2).

Microstate	D duration	D coverage
LTM	0.18	0.07

*Note*. Correlations of reliable EEG microstate measures at T1 and LTM measures (vocabulary test performance T2-T1 in %). *p < .05, **p < .01 (Bonferroni correction for multiple comparisons). Correlations are not reported for EEG microstate measures with insufficient reliability.

##### 3.2.3.1 Age-adjusted EEG-behavior associations

To further address potential age effects, age-adjusted associations between LTM performance and absolute and relative alpha power were examined in the adult sample using linear regression. After controlling for age, absolute alpha power remained a significant positive predictor of LTM change, *β* = 0.01, *t*(78) = 2.32, *p* = .023, 95% CI [0.002, 0.024], whereas age itself was not associated with LTM change, *β* < 0.001, *t*(78) = 0.07, *p* = .945.

Similarly, relative alpha power showed a significant positive association with LTM change when controlling for age, *β* = 0.87, *t*(78) = 3.43, *p* < .001, 95% CI [0.37, 1.38], while age again did not significantly predict LTM performance, *β* < 0.001, *t*(78) = −0.05, *p* = .964. These results indicate that the observed association between resting-state alpha power and LTM performance in adults is independent of age.

## 4 Discussion

Both children and adults experienced a change in LTM performance over time as measured in successful L2 learning. An overall high split-half reliability was observed for almost all measures derived from resting-state EEG. More measures of EEG power resulted in high test-retest reliability as compared to measures of microstates. Furthermore, only a few of these measures were associated with LTM performance after correcting for multiple comparisons. Higher absolute and relative alpha power at T1 were the only EEG signals positively related to a change in LTM performance from T1 to T2 in our sample and this was only observed for the adult participants. Increased alpha power at the scalp could reflect a reliable neurophysiological correlate of individual differences in LTM-related learning outcomes in the adult brain. Increased alpha power has been proposed to facilitate memory processes by suppressing unwanted or irrelevant memory traces, particularly during active memory encoding or retrieval (Hanslmayr et al., 2012). While this account is based on task-based EEG studies, our resting-state findings may reflect trait-like individual differences in the neural systems supporting such processes. Importantly, this interpretation is limited to the adult sample and does not imply that resting-state alpha power reflects a universal or developmentally invariant marker of LTM across age groups.

The high stability of the alpha band compared to other frequencies has also been noted in other studies (Popov et al., 2023). Although no direct statistical comparisons were performed between the present ICC estimates and those reported in the literature, situating our findings within the existing evidence base remains challenging due to substantial methodological heterogeneity across studies (see Lopez et al., 2023, for a review). For example, Webb et al. (2023) reported an ICC of 0.67 for absolute alpha power in a pediatric sample that was younger than the children included in the present study. In contrast, Metzen et al. (2022) observed ICC values of 0.87 and higher for absolute alpha power in adults across a wide age range (20–70 years), although these estimates were derived at the single-electrode level rather than from whole-brain averages. Similarly, Suárez-Revelo et al. (2015) reported ICC values ranging from 0.53 to 0.89 for relative alpha power in young adults (mean age ≈ 23 years), based on region-of-interest electrode analyses rather than whole-brain measures. Our results further suggest that absolute and relative alpha power are reliably related to changes in LTM performance.

Alpha power at the scalp, in general, can be interpreted as a modulation by thalamo-cortical networks (Bazanova & Vernon, 2014). Resting-state alpha power has a high heritability (Smit et al., 2006) and relates to interindividual differences in memory performance, including during tonic, task-free EEG recordings (Klimesch, 1999). Moreover, resting-state EEG metrics such as individual alpha frequency have been shown to predict individual differences in language processing during subsequent tasks (Bornkessel-Schlesewsky et al., 2022). Additionally, event-related alpha power has also been linked to lexical retrieval (Röhm et al., 2001; for a review see Bastiaansen & Hagoort, 2006; Rossi et al., 2023) and progress in L2 learning (Allal-sumoto & Duygu, 2024). While alpha power at the scalp appears to be related to LTM, intracranial recordings have linked memory consolidation to slow oscillations during non-REM sleep (Marshall et al., 2006). Other studies using intracranial recordings have linked theta-band activity during active encoding and retrieval to subsequent memory performance (for a review see Johnson & Knight, 2015). LTM-relevant brain mechanisms could manifest themselves in different frequencies at the scalp compared to intracranial level. This difference could be due to the intracranial recordings being conducted in the Hippocampus. Such oscillations are less likely to be picked up by electrodes on the scalp. One study demonstrated a correspondence between event-related intracranial and scalp EEG during successful memory encoding (Long et al., 2014). Specifically, decreases in lower frequencies (theta: 3–8 Hz) and increases in higher frequencies (gamma: 44–100 Hz) were observed to accompany recalled items in time-frequency spectrograms. However, no significant correspondence between intracranial and scalp EEG was reported for the alpha range (10–14 Hz).

Before interpreting differences between the child and adult samples, it is important to emphasize that the present study was not designed to assess developmental trajectories or to support direct statistical comparisons between age groups. Accordingly, all interpretations are restricted to age-controlled, within-sample associations rather than developmental effects across samples. Importantly, the child and adult samples in the present study differ not only in age but also in the functional role attributed to alpha oscillations in the literature. Moreover, methodological differences between study 1 and study 2 limit the interpretation of differences in outcomes as an effect of age alone. In children, lower occipital alpha power in the 7–10 Hz range during resting-state recordings has been associated with enhanced sensory and language processing, potentially reflecting reduced tonic inhibition (Kwok et al., 2019). In contrast, task-based studies in adults have linked higher left-lateralized and frontal alpha power in the 10–13 Hz range to more efficient retrieval of semantic information from LTM (Grabner et al., 2007). Although these findings derive from different paradigms and frequency ranges, such age- and context-dependent differences in alpha function may help explain the absence of significant—and directionally consistent—EEG–LTM associations in the children sample. The results in the present two studies could explain some inconsistent findings in the literature when comparing power measures and LTM based on L2 acquisition. As shown here, future studies could benefit from focusing on stable power measures, for example, alpha power. While the results in alpha power seem promising and alpha power measures are considered highly important in understanding human cognition, it is crucial to note that this resting-state and event-related power measure is not at all specific for LTM and has been associated with a wide variety of cognitive functions and impairments (Başar & Güntekin, 2012; Klimesch, 1999). LTM was the focus of the present study but any underlying cognitive process resulting in proficiency at second language acquisition could also be reflected in pronounced alpha power. For instance, LTM learning (mnemonic) and language processes (verbal) could be confounded in the present study. Future studies should try to exclude alternative cognitive processes distinguishable from LTM as explanations for the effect in alpha power. Taken together, our results suggest that higher resting-state alpha power—which likely reflects greater cortical efficiency and information processing capacity (Klimesch, 1999)—may serve as a statistically reliable predictor of LTM performance in the context of second language learning. Our findings suggest a link between resting-state alpha power and LTM improvement but we acknowledge that such interpretations must remain cautious, as resting-state EEG does not directly capture the dynamic cognitive processes (e.g., encoding, retrieval) studied in task-based memory research. While most evidence for the inhibitory role of alpha rhythms comes from task-based studies involving active suppression of distractors (e.g., Bonnefond & Jensen, 2024), such mechanisms may also be reflected in trait-like differences in resting-state alpha activity, which could underlie stable cognitive abilities like LTM performance. Resting-state alpha activity has recently been suggested to reflect not only a mechanism for specific functions such as memory consolidation, but also a flexible filter that adapts to cognitive demands—a trait-like neural feature predictive of individual differences in LTM-related abilities (Pascucci et al., 2025).

While the present results showed moderate to good reliability for some microstate measures, their relation to LTM performance appears to be nonsignificant in our samples. Microstates have sometimes been used to investigate language processes but this was mostly done with experimental tasks focusing on event-related brain potentials (Brandeis et al., 1995; Koenig et al., 1998; Koenig & Lehmann, 1996) and less in resting-state recordings like in the present study. When using lexical tasks and event-related microstates, high stability and reliability have also been reported (Laganaro, 2017), and microstates have been linked to language capabilities (Jouen et al., 2021; Tanaka et al., 2002). Such studies, however, focused less on memory and more on short-term language-specific processes, for example, speech production. Only on rare occasions were event-related microstates associated with language related stimuli (e.g. syllables) with implications on long-term cognitive processes (e.g. Giroud et al., 2017). In one such study, second language acquisition over a period of 5 months was associated with shortened task-evoked microstate durations, interpreted as reflecting faster and more efficient processing of L2 words (Stein et al., 2006). In contrast to the present study, these microstates were calculated during event-related lexical tasks rather than during resting-state recordings. In these task-based paradigms, learning-related changes in microstate dynamics were associated with shorter activation durations in regions such as the left inferior frontal gyrus. Such studies relied on structured task instructions and well-defined trial sequences, allowing learning- or language-related stimulation to be directly linked to microstate metrics. It is, therefore, possible that resting-state microstates are less sensitive to long-term memory–related changes, whereas task-evoked microstates—capturing more dynamic and context-specific neural processes—may provide greater sensitivity to learning-related performance differences.

In the present studies, microstates were calculated from resting-state recordings. As in a previous resting-state study (Antonova et al., 2022), microstate duration showed somewhat higher reliability than occurrence. However, the relation of resting-state microstates to LTM in the literature is less clear. A recent registered report with 140 healthy participants showed no significant correlation between any resting-state microstate measurements and a series of cognitive tests on executive function (e.g., letter memory task, dual N-back) (Chenot et al., 2024). Although LTM was not the focus here, executive functions rely on cognitive processes also relevant for LTM. This absence of any relation between microstate measures and cognitive performance in tasks of executive functions might also explain the absence of any such correlation in the present study. The relationship between microstates and behavioral indices of cognition could be more tangible in event-related tasks than in resting-state recordings. Another explanation could be that this relationship is more pronounced in clinical samples and not in healthy participants like in Chenot et al. (2024) or the present study. In a small-sample study, Grieder et al. (2016) found a significant correlation between resting-state microstate measures and a language test in eight patients suffering from semantic dementia. Although when using the same language test, another resting-state study with 117 patients with Alzheimer’s disease and 117 patients with mild cognitive impairment reported no significant correlation between the test scores and any microstate measure (Musaeus et al., 2020). In summary, microstates represent a very useful and easily employable analysis technique but their use in examining LTM in healthy subjects still needs further clarification. Recommendations on future directions have been pointed out (Khanna et al., 2015). At this point, the relationship between LTM and microstates may depend on the clinical characteristics of the sample, while it appears to be more pronounced when event-related task properties are employed in the experimental design.

Some notable limitations in the present study must be emphasized. Due to organizational constraints, vocabulary testing and resting-state EEG recordings could not always be conducted on the same day, and varied in timing across participants. While this introduces some temporal distance between brain and behavior measures, resting-state EEG is considered a relatively stable index of trait-like neural activity, which supports its use in examining individual differences in cognitive performance even across extended intervals. The different time intervals between T1 and T2 across the two studies (children vs. adults) may have also contributed to differences in ICC values observed between the samples. Participants going through EEG and the vocabulary test on the same day, could have experienced different impacts of fatigue or cognitive load. While handedness was recorded, it was not included as a variable in the present analyses, as we did not compute lateralized EEG indices and our reliability metrics do not accommodate covariates. This should be considered when interpreting findings, especially in the children’s sample where handedness was more variable. The different sample sizes between the studies are also quite noticeable. With more than twice the sample size in the adult study, the generally more positive results for this age-group could be explained with a simple lack of statistical power in the children group. There is also a wide age-range in the children sample. Given reported developmental changes in resting-state dynamics (e.g., Candelaria-Cook et al., 2022; Ott et al., 2021), future work should explicitly model age as a moderator of reliability. Although both children and adults were included in this study, the design does not support direct statistical comparisons between age groups. Any observed differences across groups should be interpreted with caution and not as evidence of developmental effects particularly given the opposite direction of EEG–behavior associations observed across samples. Another aspect is visualized in the behavioral results where LTM performance (T2 minus T1 in the vocabulary test) scores show a much wider distribution and high variability in children than in adults. Most adults seem to have similar changes in scores over time and a narrower distribution. This inconsistent performance in children could also be a reason why no EEG measure, despite good reliability, was significantly correlated with this behavioral measure in children. The non-perfect reliability of the LTM performance measures could have reduced our ability to find associations between EEG measures and LTM performance. Another reason for the different results in the two studies could be that children studied a somewhat familiar language while adults had to learn an entirely new language. In the children’s study, the second language tested (French/German) corresponds to one of the official national languages of Switzerland. Consequently, children likely had some prior exposure to and knowledge of this second language through their everyday environment or schooling. This uncontrolled variability in prior exposure likely contributed to the large interindividual differences in T1 performance observed in the children’s group. In contrast, in the adults’ study, the second language tested (Finnish) was entirely new to all participants, as Finnish is neither an official language of Switzerland nor a language to which participants had prior exposure. Language familiarity alone may influence motivational factors (Liu, 2025) and has also been shown to affect EEG parameters during language tasks (de León Rotriquez et al., 2022; Ihara et al., 2021), which could have contributed to differences between the two samples in our study. Even when resting-state recordings make it easier to study children with EEG than event-related tasks, the vocabulary testing sessions are still prone to commonly associated problems when doing research with children. While new methods for resting-state EEG analysis are constantly being developed, sometimes specifically within language research (Roehm et al., 2004), there is also a need to identify sources for the heterogenous results in the existing literature and establish metrics of reliability within a field for it to move forward.

### 4.1 Conclusion

This study set out to test the stability of two very promising resting-state EEG methodologies by calculating both test-retest and split-half reliabilities. Based on the present results, power values produce mostly, and microstates produce some stable measures. Future inconsistent findings could be avoided by focusing more on the stable measures within these two EEG methodologies. Despite their stability, their exact relationship with LTM remains a challenging question. Out of all the identified stable measures, only alpha power appears to be linked to LTM and only in adult participants. This finding, if replicated, should be evaluated using different approximations to LTM than L2 vocabulary tests and a larger children sample.

## Data Availability

All data and analysis scripts used in this study are publicly available at the Open Science Framework (OSF): https://osf.io/5znyk/, DOI: https://doi.org/10.17605/OSF.IO/5ZNYK.
